# A multi-tissue transcriptomic landscape of female mice in estrus and diestrus provides clues for precision medicine

**DOI:** 10.3389/fcell.2022.983712

**Published:** 2022-12-16

**Authors:** Yiran Zhou, Han Yan, Wenjun Liu, Chengqing Hu, Yuan Zhou, Ruya Sun, Yida Tang, Chao Zheng, Jichun Yang, Qinghua Cui

**Affiliations:** ^1^ Department of Biomedical Informatics, Center for Noncoding RNA Medicine, MOE Key Lab of Cardiovascular Sciences, School of Basic Medical Sciences, Peking University, Beijing, China; ^2^ Department of Physiology and Pathophysiology, Center for Noncoding RNA Medicine, MOE Key Lab of Cardiovascular Sciences, School of Basic Medical Sciences, Peking University, Beijing, China; ^3^ Department of Medicinal Chemistry, School of Pharmaceutical Sciences, Peking University, Beijing, China; ^4^ Department of Endocrinology, The Second Affiliated Hospital, School of Medicine, Zhejiang University, Hangzhou, China; ^5^ Department of Cardiology, MOE Key Lab of Cardiovascular Sciences, Peking University Third Hospital, Beijing, China

**Keywords:** reproductive cycle, estrous cycle, systems biology, RNA-sequencing, hypertension

## Abstract

Female reproductive cycle, also known as menstrual cycle or estrous cycle in primate or non-primate mammals, respectively, dominates the reproductive processes in non-pregnant state. However, in addition to reproductive tissues, reproductive cycle could also perform global regulation because the receptors of two major female hormones fluctuating throughout the cycle, estrogen and progesterone, are widely distributed. Therefore, a multi-tissue gene expression landscape is in continuous demand for better understanding the systemic changes during the reproductive cycle but remains largely undefined. Here we delineated a transcriptomic landscape covering 15 tissues of C57BL/6J female mice in two phases of estrous cycle, estrus and diestrus, by RNA-sequencing. Then, a number of genes, pathways, and transcription factors involved in the estrous cycle were revealed. We found the estrous cycle could widely regulate the neuro-functions, immuno-functions, blood coagulation and so on. And behind the transcriptomic alteration between estrus and diestrus, 13 transcription factors may play important roles. Next, bioinformatics modeling with 1,263 manually curated gene signatures of various physiological and pathophysiological states systematically characterized the beneficial/deleterious effects brought by estrus/diestrus on individual tissues. We revealed that the estrous cycle has a significant effect on cardiovascular system (aorta, heart, vein), in which the anti-hypertensive pattern in aorta induced by estrus is one of the most striking findings. Inspired by this point, we validated that two hypotensive drugs, felodipine and acebutolol, could exhibit significantly enhanced efficacy in estrus than diestrus by mouse and rat experiments. Together, this study provides a valuable data resource for investigating reproductive cycle from a transcriptomic perspective, and presents models and clues for investigating precision medicine associated with reproductive cycle.

## 1 Introduction

Reproductive cycle is a typical feature of female placental mammals within reproductive period, which comprises a periodic series of physiological changes caused by fluctuant hormones, especially estrogen and progesterone. For most non-primate mammals such as mice, rats, dogs, pigs and so on, reproductive cycle is also known as estrous cycle and accepted to be divided into four sequential phases including proestrus, estrus, metestrus and diestrus (Spornitz et al., 1994; Concannon, 2011; Soede et al., 2011; Gal et al., 2014; Ajayi and Akhigbe, 2020). During the estrous cycle, the proestrus-estrus junction is featured with strong stimulus of estrogen but the minimal impact of progesterone whereas the diestrus phase is characterized by a high level of progesterone but basal concentration of estrogen. As for the primates (i.e., human beings and the great apes), reproductive cycle is well-known as menstrual cycle (Mihm et al., 2011). However, in terms of cyclic hormonal fluctuation and ovulation, the menstrual cycle is homologous to the estrous cycle.

The cyclic hormonal milieu precisely controls the endometrium morphology. However, in addition to the uterus, estrogen/progesterone receptor are also distributed in a wide range of non-reproductive tissues (Karas et al., 1994; Hogan et al., 2009; Tetel and Pfaff, 2010; Barros and Gustafsson, 2011; Iorga et al., 2018; Asavasupreechar et al., 2020). Therefore, reproductive cycle is not only responsible for reproduction but could also regulate many other biological processes. For example, Chen et al. reported that the rats in estrus are more sensitive to fear than those in diestrus (Chen et al., 2009), suggesting the altered neural activities. Recent studies also showed the difference of inflammatory responses between estrus mice and diestrus mice (Islam et al., 2016; Fuentes et al., 2019), revealing the remodeled immunity. So far, the outcomes of many tissues under exogenous hormonal treatment have been well-studied (Barros and Gustafsson, 2011; Bosch et al., 2018). However, their alterations in the real reproductive cycle, where the hormone levels are more natural, dynamic and complicated, have not yet been fully understood to our best knowledge. Moreover, even though some phenotypes associated with real reproductive cycle have been reported (Chen et al., 2009; Hogan et al., 2009; Saito et al., 2009), the underlying molecules are still required to be systematically profiled. Overall, many important questions still remain to be solved such as during the reproductive cycle which tissues are prominently influenced? How is the molecular regulatory network altered in individual tissues? Which biological processes are the regulated molecules involved in? And how could the systemic changes guide us in developing precision medicine strategies towards pre-menopausal women?

**FIGURE 1:**
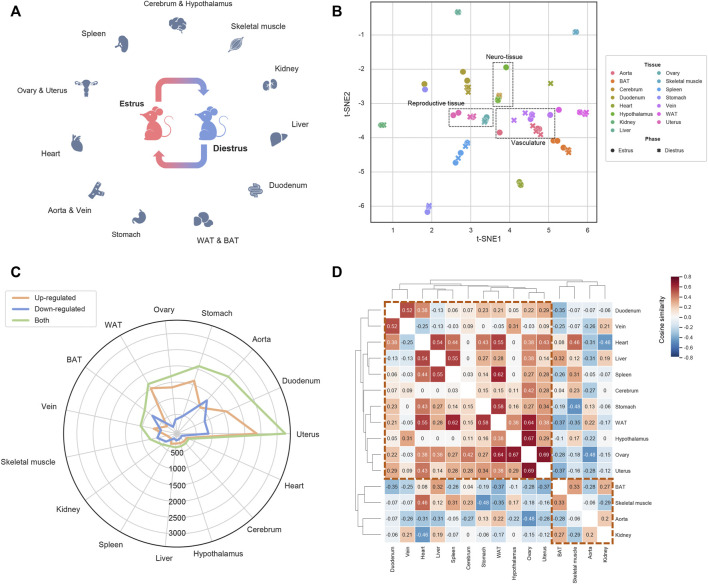
Overview of the transcriptomic landscape under estrous cycle **(A)** The transcriptomic landscape covering 15 tissues dissected from the mice at the estrus phase and the diestrus phase. For each tissue-phase group three biological replicates were set, resulting in 90 samples in total **(B)** Sample visualization by t-SNE based transcriptomic dimensionality reduction **(C)** The DEG number of each tissue **(D)** The tissue clustering map based on the cosine similarity of the shared DEGs’ alteration trends.

In this study, we monitored the estrous cycles of C57BL/6J mice according to the patterns of vaginal epithelial stratification and serum hormone levels. Considering estrus and diestrus are two typical phases in estrous cycle, which were often visited in previous researches (Skulska et al., 2019; An et al., 2021; Bernhardt et al., 2021), we established a transcriptomic landscape across 15 tissues in estrus and diestrus (Figure 1A) by RNA-sequencing. Based on the gene expression data, we firstly uncovered a number of genes, pathways and transcription factors involved in the estrous cycle on a multi-tissue scale. Then, we evaluated the physiological changes, potential risks and benefits caused by estrus and diestrus for individual tissues using bioinformatics modeling. Inspired by the anti-hypertensive transcriptomic pattern induced by estrus in aorta, enhanced efficacy of hypotensive drugs at the estrus phase were experimentally validated in both mouse and rat models in the end.

## 2 Materials and methods

### 2.1 Experimental animals and monitoring of estrous cycle

Female C57BL/6J mice and Sprague Dawley rats aged 8 weeks were used in the study. All animals were housed separately in a standard laboratory environment with a 12-h dark-light cycle and had free access to food and water. The estrous cycle of female mice and rats lasts for 4–5 days, and undergoes four sequential phases including proestrus, estrus, metestrus and diestrus. In this work, a vaginal cytology method was applied to distinguish these phases. As previously described (Ajayi and Akhigbe, 2020; Wang et al., 2020), animals’ vaginal secretion was collected first by micropipettes (the tips were filled with a small amount of 0.9% saline), and then the flushed fluid was placed on glass glides. Next, the glass slides were dried and stained with 0.1% methylene blue (Sigma) for 15 min and washed by PBS for two times so that the estrous cycle phases could be microscopically identified based on the vaginal epithelial cellular types and proportions (Supplementary Figures S1,S2). For each animal, the estrous cycle was daily monitored in the morning for at least 2 weeks to precisely trace the individual-specific period. In addition, the concentrations of serum estrogen and progesterone of mice were also measured to assist the determination of estrous cycle phases. Specifically, the supernatant of internal canthus blood of mice in estrus or diestrus was first collected after homogenization for 20 min (1,000 × g). Then, the serum estrogen and progesterone levels were quantified using the Elisa Kits (#JL 11232, Jianglai Biotechnology and #E-EL-0154c, Elabscience), at a wavelength of 450 nm. As expected, the levels of estrogen and progesterone are indeed significantly higher in estrus and diestrus, respectively (Supplementary Figure S3).

### 2.2 RNA extraction and high-throughput RNA-sequencing

Normal female C57BL/6J mice in estrus and diestrus were sacrificed in triplicate to harvest 15 tissues including aorta, vein, brown adipose tissue, white adipose tissue, heart, kidney, liver, skeletal muscle, stomach, duodenum, cerebrum, hypothalamus, ovary, uterus and spleen. The collected tissues were rapidly frozen on dry ice. Total RNA was extracted using Trizol (Invitrogen, Carlsbad, CA, United States) and further qualified and quantified using a Nano Drop and Agilent 2100 bioanalyzer (Thermo Fisher Scientific, MA, United States). The RNA-sequencing libraries were constructed by the TruSeq RNA Library Prep Kit v2 and the reads were sequenced by the BGISEQ-500 platform (Bgi Genomics Co., Ltd). For each sample, approximately 22M reads with a high average quality score of 37 were generated. The data had been uploaded to the CNCB-NGDC GSA database (CRA004295) and the NCBI GEO database (GSE131172).

### 2.3 Identification and analysis of differentially expressed genes

For the raw sequencing data, the software SOAPnuke (Chen et al., 2017) was firstly used to remove the adaptor-contaminated and low-quality reads. Subsequently, the retained reads with high-quality were aligned to the GRCm38 genome (NCBI version p5) by Bowtie2 (Langmead and Salzberg, 2012) with the parameters of “--sensitive --dpad 0 --gbar 99999999 --mp 1,1 --np 1 –score-min L,0,-0.1 –k 200”. The resulting bam files were further inputted into RSEM (Li and Dewey, 2011) to quantify the gene expression abundance. Then for each tissue, differentially expressed genes (DEGs) between estrus and diestrus were identified by the R package DEGSeq (Wang et al., 2010), with the criterion of |log_2_ (fold change)| ≥ 1 and q-value ≤0.05. Finally, the DEGs were mapped to human orthologous genes (hDEGs) by the g:Profiler platform (Raudvere et al., 2019).

We conducted three analyses for the hDEGs: 1) Traditional functional enrichment analysis. The gene sets of Kyoto Encyclopedia of Genes and Genomes (KEGG pathway), Molecular Signatures Database Hallmark (MSigDB Hallmark) and Genetic Association Database (GAD) were mainly concerned, and the statistical calculation (Fisher exact test and *p*-value correction) was implemented by the R package clusterProfiler (Yu et al., 2012). 2) Network centrality analysis. Briefly, we introduced a manually curated human molecular signaling network (downloaded from http://www.cancer-systemsbiology.org) and studied whether the hDEGs are of higher hubness (measured by network centrality indices) than background. The construction of directional network was implemented by the python package NetworkX, in which genes and interactions were modeled as nodes and edges, respectively. Nodes’ centrality indices including degree, betweenness and PageRank were calculated *via* NetworkX as well. Finally, one-sided Wilcoxon rank-sum test was used to measure the centrality differences between hDEGs and background. 3) Causal inference of functional transcription factors (TFs). In this analysis, we took advantage of an online platform, CIE, which is designed for detecting functional TFs (i.e., activity-up or -down TFs) by measuring the consistency between the inputted transcriptomic alteration profile and its built-in TF-gene regulation relationships (Farahmand et al., 2019). Here the functional TFs related to estrous cycle were determined by the CIE quaternary test (one sided q-value ≤0.05; Supplementary Data S1).

### 2.4 Manually curated transcriptomic alteration signatures and signature comparative analysis

By querying tissue names in the GEO database, we manually collected a considerable number of transcriptomic cases of mouse models under various physiological and pathological states such as disease modeling, drug treatment, lifestyle intervention and so on. For each case, a log_2_ (fold change)-based transcriptomic alteration signature (TAS) was calculated by comparing the gene expression profiles between experimental samples and control samples. Next, pairwise Spearman’s correlation coefficients (SCCs) among these TASs were calculated and the redundant TASs (SCC ≥0.5) were rationally removed. We also labeled the cases by their outcomes (i.e., “beneficial” and “deleterious”) and experiment types. For example, the ovary case ‘Ovarian tumor, Arid1a/Pten knockout model’, in which ovarian tumor samples resulting from Arid1a/Pten double-knockout were compared to normal ovaries, was labeled as ‘deleterious, disease, cancer’. For another example, the liver case “Type 2 diabetes (db/db model), rescued by FGF19”, where db/db mice treated with exogenous FGF19 exhibited promoted HDL biogenesis relative to control, was annotated with “beneficial, chemical treatment”. As a result, we curated 1,263 TASs across 15 tissues in total. The detailed information of the curated TASs is listed in the Supplementary Data S2.

In the TAS comparative analysis, we first calculated log_2_ (fold change)-based estrus-induced TAS for each tissue by comparing estrus with diestrus, based on the transcriptomic data generated in this work. Then, SCCs between the estrus-induced TASs and the curated TASs from the same tissue origin were calculated so that the changes of individual tissues brought by estrus and diestrus could be inferred from the significantly correlated relationships (defined as |SCC| ≥ 0.1). It should be noted that the SCC-based cutoff may introduce slight biases when the curated TASs are of different dimensionalities, but here we aimed to discover informative patterns from the estrus-induced TASs rather than strictly ranking the correlated relationships. If an obvious beneficial or deleterious tendency could be commonly reflected by the correlated TASs according to the manually curated labels of “beneficial” and “deleterious” mentioned above, a reliable protective or harmful role of estrus relative to diestrus could be rationally inferred.

### 2.5 Treatment of hypotensive drugs and measurement of blood pressure

Felodipine (ab141797, Abcam) was given orally at a dose of 5 mg/kg body weight for mice, and 30 mg/kg for rats. Acebutolol (#15372, MedChemExpress) was given at a 2 mg/kg oral dose for mice, and 50 mg/kg for rats, respectively. The doses were determined based on the previous researches (Basil et al., 1974; Mostafavi and Foster, 2003; Sridhar et al., 2014; Lam et al., 2018). The drug administration was conducted on both hypertensive and normotensive rodent (mouse and rat) models. The spontaneously hypertensive female rats (SHR) were purchased from the Charies River Company and the hypertensive female mice were constructed by 2-week angiotensin-II (#H-1706, Bachem, 1,000 ng/kg/min) infusion using Alzet osmotic pumps 2004 (Alzet, United States). After single dose of drug gavage, animals’ blood pressure levels were measured in the following 8 h using a non-invasive tail cuff system, which consists of an inflatable tail cuff and an electronic blood pressure meter (Kent, United States) (Seto et al., 2013; Wang et al., 2017). For each time of measurement, the rodents were first placed in a 37°C temperature for 5 min and then pre-tested for 5 times to adapt the environment. Next, the blood pressure levels were formally tested for 10 times, and the mean values were finally recorded. All animal treatments were approved by the Ethics Committee of Peking University Health Science Center.

## 3 Results

### 3.1 Overview of the transcriptomic landscape during estrus and diestrus

To understand the transcriptomic dynamics during the estrous cycle, we selected two most representative phases, estrus and diestrus, and for each phase we assigned three mice as biological replicates, of which samples from 15 tissues were dissected (aorta, vein, brown adipose tissue, white adipose tissue, heart, kidney, liver, skeletal muscle, stomach, duodenum, cerebrum, hypothalamus, ovary, uterus and spleen; Figure 1A). Transcriptomes of the total 90 samples were profiled by RNA-sequencing technique. Based on the resulting gene expression profiles, t-distributed stochastic neighbor embedding (t-SNE) was firstly applied to visualize the sample similarities and differences (Figure 1B). As a result, we observed some functionally related tissues, such as aorta and vein, ovary and uterus as well as cerebrum and hypothalamus, tend to be adjacent with each other. We also noticed the samples from the same tissue but different phases are less distributed than those from the same phase but different tissues. Therefore, beyond the different phases, tissues’ inherent biological characteristics could be more contributive to the sample divergence. Then by comparing estrus with diestrus, we identified differentially expressed genes (DEGs) for each tissue. Figure 1C shows the numbers of up- and down-regulated DEGs of each tissue, where aorta (989 up-regulated *vs.* 1,288 down-regulated DEGs), brown adipose tissue (BAT; 291 up *vs.* 848 down), kidney (151 up *vs.* 327 down) and skeletal muscle (221 up *vs.* 530 down) exhibit more down-regulated DEGs, whereas the other tissues showed more up-regulated DEGs. Moreover, uterus, duodenum, aorta, stomach, ovary and white adipose tissue (WAT) have much more DEGs than the other tissues, therefore these six tissues are prioritized as the target tissues of estrous cycle (ECTTs) in this study. Subsequently, we calculated cosine similarities for all pairs of tissues based on the alteration trends of the shared DEGs (up- and down-regulation were represented by +1 and −1 respectively; Figure 1D). As shown in Figure 1D, tissues are macroscopically clustered into two modules which perfectly match the tissue groups enriched with up- and down-regulated DEGs mentioned above, indicating that at the transcriptional level, the estrous cycle could differentially regulate the hDEGs shared by different subsets of tissues.

### 3.2 Comprehensive analyses of differentially expressed genes

**FIGURE 2:**
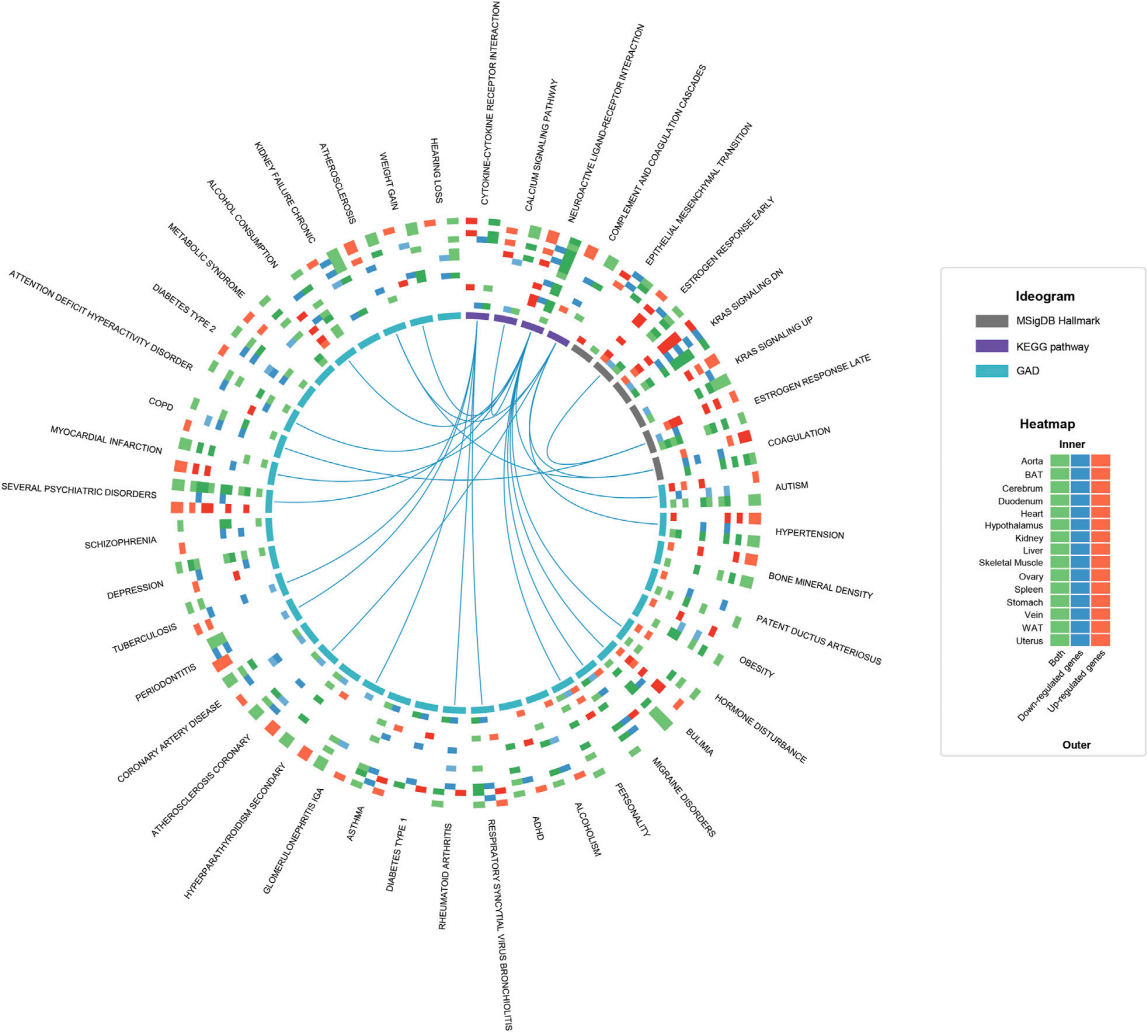
Functional enrichment analysis of hDEGs on a multi-tissue scale. The circos plot shows the statistically significant terms (q-value ≤0.05) shared by at least five tissues in the functional enrichment results of KEGG, MSigDB Hallmark and GAD. For each term, a heatmap with 15 rows and 3 columns is plotted, where the rows represent tissues and the columns represent up-regulated hDEGs (red), down-regulated hDEGs (blue) and both (green), respectively. The colored cells mean that the corresponding hDEG sets are significantly functionally related to the term, and the color depth reflects the statistical significance. The term-term links mean that the corresponding gene sets are significantly overlapped (defined as sharing no less than 25% hDEGs). To avoid over-redundancy, the links between GAD terms are hidden.

To deeply understand the influences of estrous cycle, we firstly carried out functional enrichment analysis towards hDEGs (human orthologous genes of DEGs) for each tissue, referring to the gene sets of Kyoto Encyclopedia of Genes and Genomes (KEGG pathway), Molecular Signatures Database Hallmark (MSigDB Hallmark) and Genetic Association Database (GAD). The statistically significant terms shared by at least five tissues are further screened (Figure 2). Firstly, several biological processes were underlined by the KEGG and MSigDB Hallmark results. For example, in line with the fluctuant levels of estrogen between estrus and diestrus, the MSigDB Hallmark gene sets about estrogen response are overrepresented in 10 tissues (aorta, BAT, cerebrum, duodenum, heart, skeletal muscle, ovary, stomach, vein and uterus), consistently most of which have been reported serving as estrogen target tissues (Karas et al., 1994; Hogan et al., 2009; Tetel and Pfaff, 2010; Barros and Gustafsson, 2011; Iorga et al., 2018; Ajayi and Akhigbe, 2020), indicating the widespread influences of estrous cycle. In addition, the gene sets about complement and coagulation cascades were identified in seven tissues (duodenum, hypothalamus, ovary, stomach, vein, WAT and uterus). It has been reported that the blood coagulation can be remodeled in the uterus during the estrus/menstrual cycle (Ruiz-Alonso et al., 2012; Yip et al., 2013), thus combining with our result, this phenomenon could be generalized to multiple tissues. We also observed “Neuroactive ligand-receptor interaction” and “Cytokine-cytokine receptor interaction” in 12 tissues (tissues except for BAT, heart and liver) and five tissues (aorta, duodenum, stomach, vein and uterus) respectively, demonstrating the widely neuro- and immuno-regulatory roles of estrous cycle. The KRAS pathway and the calcium signaling pathway were also presented in multiple tissues, but further experimental validation is required due to insufficient publications. As for the GAD results, we summarized three main categories of diseases that may be associated with estrous cycle, namely psychiatric disorders, inflammation and cardiovascular diseases (CVDs), all of which are closely associated with the pathways discussed above (links in Figure 2). The KEGG and MSigDB Hallmark results of individual tissues are shown in Supplementary Figures S4,S5.

Next, we investigated whether the hDEGs tend to serve as “hub genes” in intricate biological pathways. Specifically, we mapped the hDEGs onto a manually curated molecular signaling network and calculated three centrality indices including degree, betweenness and PageRank for them to quantify the hubness (Supplementary Figure S6). Surprisingly, except for the vasculature, almost no tissue is enriched with high-hubness hDEGs. What’s more, the centrality of stomach’s and uterus’s hDEGs is even significantly lower than the background. Therefore, we inferred that estrous cycle may just affect tissues in a moderate way, even for most ECTTs (uterus, stomach, ovary, duodenum and WAT). In other words, during the estrous cycle, the body functions are prone to keep stable instead of being radically altered. But the vascular tissues, especially the aorta, could be profoundly influenced differing from the other ECTTs.

**FIGURE 3:**
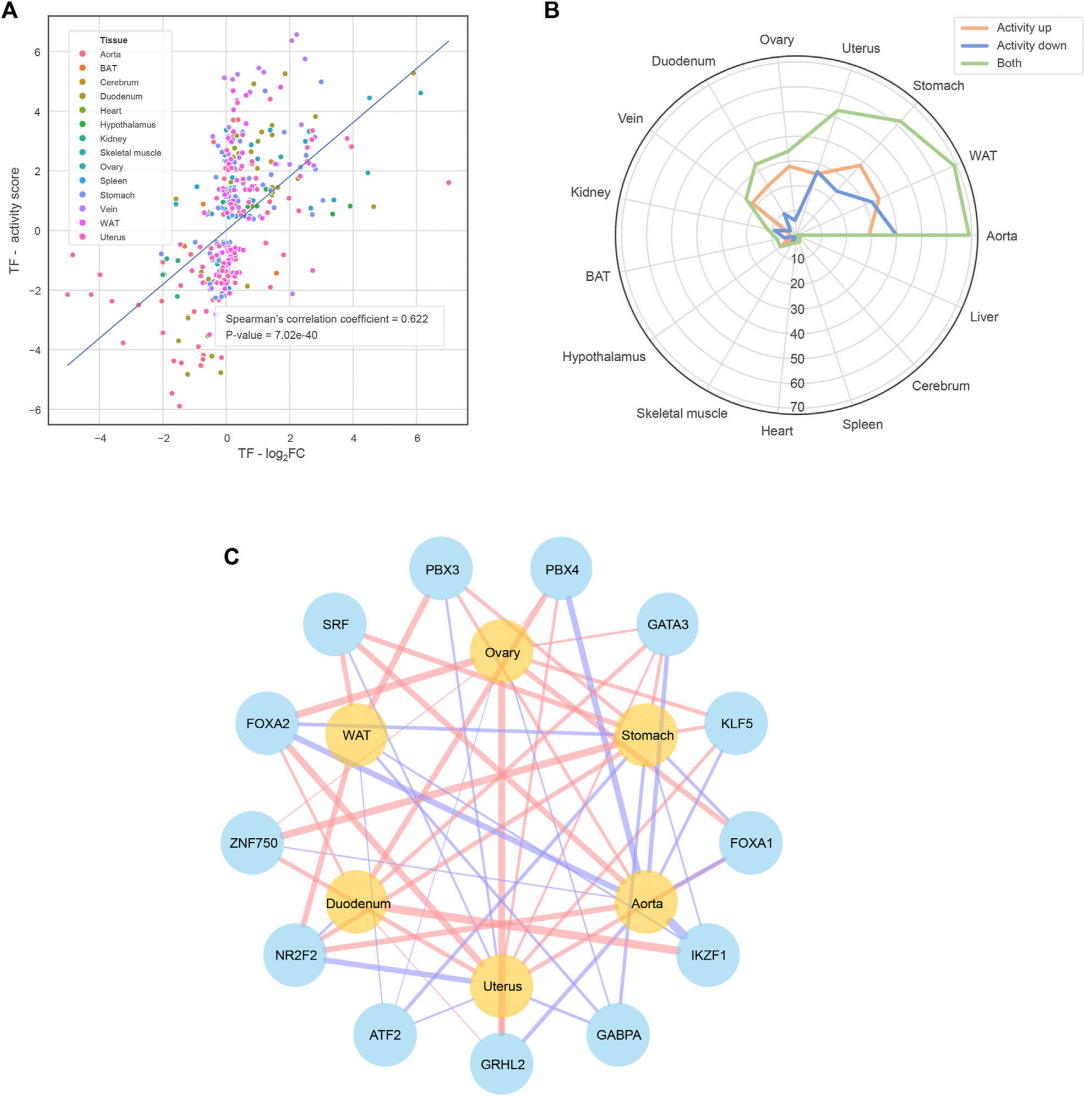
Functional TFs identified by the CIE tool **(A)** The strong positive correlation between the functional TFs’ expression abundance fold change and downstream transcriptional regulation activity. The transcriptional regulation activity is quantified by shrunk CIE q-value, namely the activity score of the *y*-axis **(B)** The number of functional TFs of each tissue **(C)** The TFs shared by at least four ECTTs. The red/blue TF-tissue links mean that the TFs exhibit significantly increased/decreased activity in corresponding tissues when comparing estrus *versus* diestrus and the link width shows the activity score defined in the subplot **(A)**

To investigate the mechanism behind the transcriptomic alteration, we further performed a causal inference analysis of functional transcription factors (TFs) using the CIE tool (Farahmand et al., 2019). Taking the alteration trends of hDEGs as input, CIE returned the TFs predicted with significantly increased or decreased activity at the estrus phase for individual tissues (Supplementary Data S1). As expected, the expression fold change of these functional TFs is strongly positively correlated with their activity of downstream transcriptional regulation (Spearman’s correlation coefficient = 0.622, *p*-value = 7.02e-40; Figure 3A). Furthermore, we found the TFs tend to act in a tissue-specific manner, that is to say, despite the same TF could be oppositely regulated in different tissues (Supplementary Data S1), which may partly explain the phenomenon described in Figure 1D. We also noticed that the ECTTs exhibit much more functional TFs in comparison with the other tissues (Figure 3B). Therefore, we focused on the ECTTs and identified 13 TFs shared by at least four ECTTs as the key TFs involved in estrous cycle (Figure 3C). Accordantly, most of these key TFs, including ATF2, NR2F2, FOXA1, FOXA2, GATA3, KLF5, SFR and GRHL2, have been shown to interact with estrogen/progesterone receptor signaling pathways (Guo et al., 2010; Liu et al., 2011; Chi et al., 2019; Fitzgerald et al., 2019; Jiang et al., 2019; Giannoudis et al., 2020; Guo et al., 2020; Wu et al., 2020; Zhou et al., 2020), reflecting their important roles in transcriptional regulation during the estrous cycle.

### 3.3 Comparative analysis of transcriptomic alteration signatures characterizes the effects of estrus and diestrus on individual tissues

**FIGURE 4:**
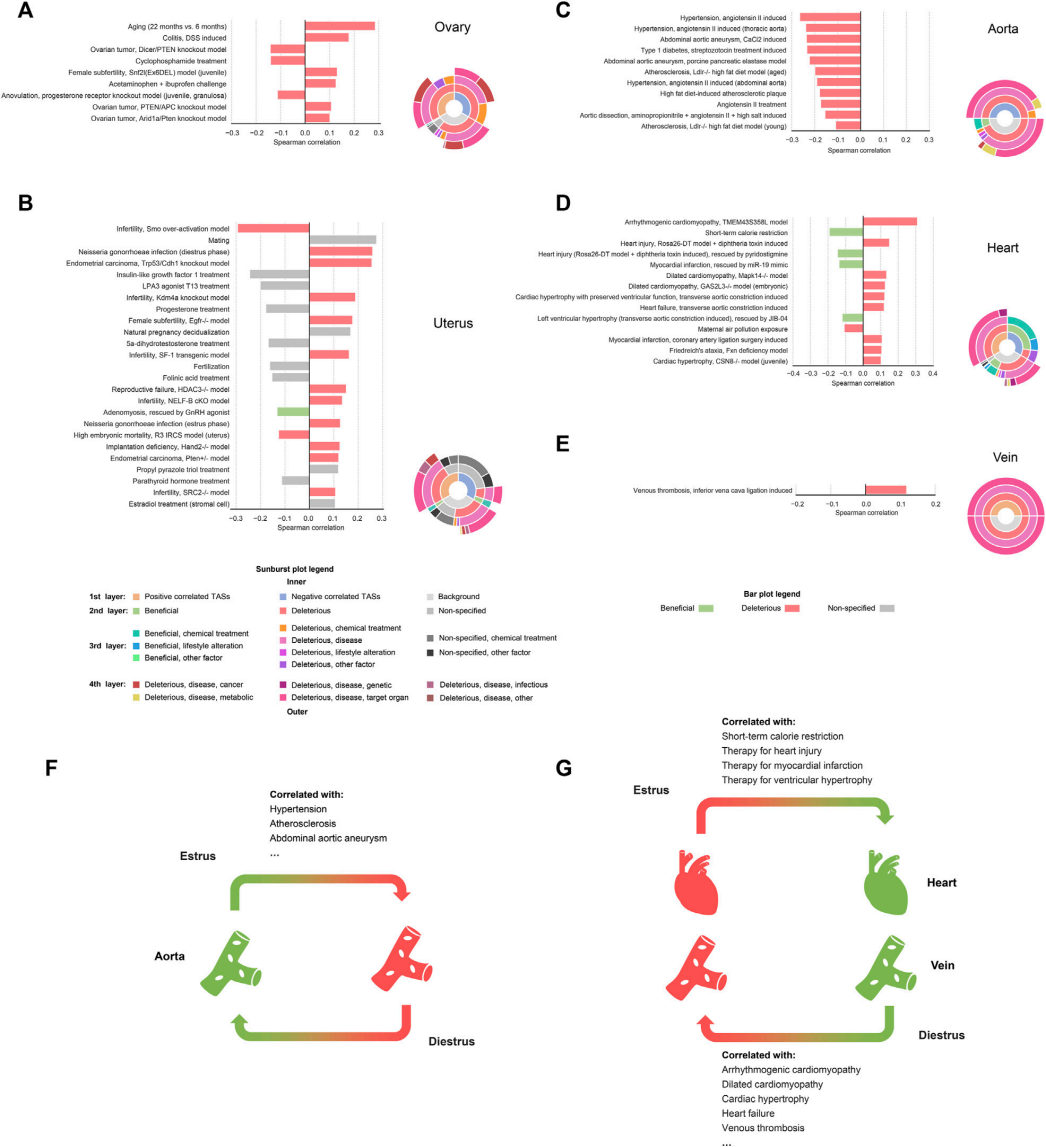
Significantly correlated relationships between estrus-induced TASs and curated TASs in reproductive tissues and cardiovascular tissues. Significantly correlated relationships in reproductive tissues and cardiovascular tissues **(A)** ovary **(B)** uterus **(C)** aorta **(D)** heart and **(E)** vein. Bar plots illustrate the curated TASs that are significantly correlated with the estrus-induced TASs in corresponding tissues (|SCC| ≥ 0.1). Sunburst plots show the hierarchical affiliation relationships among the multi-level labels of the positively correlated TASs, the negatively correlated TASs and the background. During the estrous cycle **(F)** the estrus phase is more protective to aorta while **(G)** the diestrus phase is more protective to heart and vein.

To further characterize the effects brought by estrus/diestrus on each tissue, we introduced a computational approach based on the transcriptomic alteration signature (TAS), which is defined as log_2_ (fold change)-based array that profiles the whole transcriptomic alteration. Briefly, we manually curated and annotated 1,263 TASs of various physiological and pathophysiological states. Then, we conducted a TAS comparative analysis where the Spearman’s correlation coefficients (SCCs) between the estrus-induced TASs and the curated TASs from the same tissue origin were calculated so that the physiological changes and beneficial/deleterious effects brought by estrus/diestrus could be rationally inferred (See “Materials and Methods” for detail). In this section we emphasize on the results of reproductive organs (ovary and uterus) and cardiovascular system (aorta, vein and heart) (Figure 4).

The TAS comparative results of ovary and uterus provided some valuable information (Figures 4A, B). Firstly, there is a positive correlation between the estrus-induced TASs and the disease models’ TASs of infertility caused by luteinization failure (Snf2l-Ex6DEL model: SCC = 0.131, *p*-value = 2.96e-61) or decidualization failure (Kdm4a knockout model: SCC = 0.189, *p*-value = 1.01e-122; Egfr−/− model: SCC = 0.177, *p*-value = 5.02e-109; SF-1 transgenic model: SCC = 0.163, *p*-value = 1.29e-91; Hdac3−/− model: SCC = 0.151, *p*-value = 3.64e-79). During the estrus-diestrus transition, the corpus luteum is newly formed in ovary and begins to secret abundant progesterone. Meanwhile under the action of progesterone, endometrium is remodeled to get ready for pregnancy (Billhaq et al., 2020). Thus, both ovary and uterus have more fertile characteristics in diestrus than estrus, indirectly supporting the positive correlation between estrus and infertility. However, as for the infertile model induced by Smo over-activation, a negative relationship was observed (SCC = -0.294, *p*-value < 1e-300). Unlike the other ones, this model leads to infertility not by directly disturbing the luteinization or the decidualization, but by deforming the uterus morphology *via* excessively amplifying the hedgehog signaling (Franco et al., 2010). In our data, however, a weakened hedgehog signaling was witnessed in uterus when comparing estrus with diestrus, where several upstream genes including Ihh, Dhh, Ptch1, *P*th2 and Gli1 are all significantly down-regulated, resulting in the exceptional negative correlation. The estrus-induced TASs also exhibit a positive relationship with mating behavior (SCC = 0.275, *p*-value = 1.65e-163) and a negative relationship with anovulation (SCC = -0.113, *p*-value = 3.67e-48) respectively in uterus and ovary, in line with the phenomenon of mating receptivity and ovulation at the estrus phase. Interestingly, the estrus-induced TASs are also found to be positively correlated with inflammatory and infectious TASs such as colitis (SCC = 0.179, *p*-value = 2.62e-291) and gonorrheal infection (SCC = 0.259, *p*-value = 1.09e-254), which may relate to the inflammation-like responses during the peri-ovulatory period, where both follicles and uterus are infiltrated with leukocytes (Westwood, 2008; Duffy et al., 2019). There are also several meaningful TASs about hormonal treatment presented in uterus. Specifically, in accordance with the strong stimulus of estrogen at the estrus phase, the treatment of propyl pyrazole triol, an estrogen selective to estrogen receptor alpha (SCC = 0.119, *p*-value = 2.81e-49), and estradiol, the major type of estrogen (SCC = 0.106, *p*-value = 2.25e-41), are both positively associated with estrus. By contrast the progesterone treatment shows a negative correlation as expected (SCC = -0.178, *p*-value = 2.32e-58). Besides, the administration of LPA3 agonist T13 (SCC = -0.201, *p*-value = 6.23e-132) and five alpha-dihydrotestosterone (SCC = −0.168, *p*-value = 3.35e-52), which are capable of synergizing with progesterone (Hama et al., 2006; Babayev et al., 2017), are also negatively correlated with estrus. It should be noted that even though many correlated TASs mentioned above are labeled with “deleterious”, they may just reflect the physiological differences of reproductive tissues between estrus and diestrus rather than real harms in consideration of reproductive tissues’ particularity. Nevertheless, some other correlated TASs may be indicative of potential risks brought by estrus. For instance, ovarian cancer (Pten/Apc knockout model: SCC = 0.107, *p*-value = 9.35e-41; Arid1a/Pten knockout model: SCC = 0.101, *p*-value = 2.71e-24) and endometrial cancer (Trp53/Cdh1 knockout model: SCC = 0.256, *p*-value = 1.03e-191; Pten ± model: SCC = 0.120, *p*-value = 2.92e-39) were found to be positively associated with estrus. Indeed, the increased number of ovulations is widely considered as a risk factor of reproductive cancers (Purdie et al., 2003; Reis and Beji, 2009; Zucchetto et al., 2009; Ali, 2013; Trabert et al., 2020), where more ovulations mean more exposure to proinflammatory environment for ovary and proliferous stimulus for uterus. We also discovered an indication of aging from the estrus-induced TAS of ovary (SCC = 0.286, *p*-value = 2.62e-291), which may be connected with the declining ovarian function along with incessant ovulations.

As for the cardiovascular tissues, namely aorta, heart, and vein, we observed obvious beneficial and deleterious tendencies from the correlated TASs (Figures 4C–E). Specifically, in aorta, the estrus-induced TAS is strongly negatively correlated with the TASs of disease models of hypertension (SCC = -0.265, *p*-value = 8.78e-121), atherosclerosis (SCC = -0.199, *p*-value = 8.41e-114), abdominal aortic aneurysm (CaCl2 induced: SCC = -0.236, *p*-value = 1.41e-182; porcine pancreatic elastase model: SCC = −0.223, *p*-value = 1.62e-161) and so on, indicating that estrus is more beneficial to aorta relative to diestrus (Figure 4F). In consideration of the strong stimulus of estrogen at the estrus phase, this result is in line with the vascular protective effects of estrogen proposed by many studies (Ailawadi et al., 2004; Xue et al., 2007; Meng et al., 2021). But surprisingly, contrary to aorta, relatively deleterious effects of estrus were observed in heart, where the TASs of cardiopathy models such as arrhythmogenic cardiomyopathy (SCC = 0.309, *p*-value = 8.20e-274), dilated cardiomyopathy (Mapk14−/− model: SCC = 0.134, *p*-value = 5.26e-57; GAS2L3−/− model: SCC = 0.126, *p*-value = 1.01e-50), cardiac hypertrophy (SCC = 0.123, *p*-value = 3.24e-52) and so on clearly positively correlate with the estrus-induced TAS, while the TASs of therapies towards heart injury (SCC = -0.146, *p*-value = 1.05e-73), myocardial infarction (SCC = -0.137, *p*-value = 9.98e-66) and left ventricular hypertrophy (SCC = -0.119, *p*-value = 5.76e-46) show negative correlation. Moreover, the only one TAS curated for vein, namely venous thrombosis (SCC = 0.118, *p*-value = 4.85e-50), is positively correlated with estrus, exhibiting relatively deleterious implication as well (Figure 4G). In fact, although some cardiovascular protective mechanisms of estrogen have been revealed (Mahmoodzadeh et al., 2014; Pedram et al., 2016), unopposed estrogen treatment also controversially shows risks of arrhythmia, sudden cardiac death and venous thrombosis in animal experiments and hormonal therapy studies (Canonico et al., 2007; Odening et al., 2012; Yan et al., 2013), which are in high agreement with our results. What’s more, the relatively deleterious implication of estrus on heart and vein also means the relatively beneficial implication of diestrus. Indeed, as the most representative hormone of diestrus, progesterone is also shown to be protective to cardiovascular system (Thomas and Pang, 2013) and more importantly, likely to overcome the side effects of estrogen (Tchaikovski and Rosing, 2010; Odening et al., 2012), further supporting our results. In addition, through the TAS comparative analysis, BAT and stomach are also observed getting more benefits from the estrus, while liver, kidney, skeletal muscle and WAT receive more protection from the diestrus (Supplementary Material; Supplementary Figures S7,S8). That is to say, estrus and diestrus could exert asynchronous protective influences on different subsets of tissues, respectively. In other words, there exists a phenomenon of phase-to-tissue specific protection (PTSP) during the estrous cycle.

The PTSP phenomenon also indicates that specific phases of estrous cycle could induce disease-resistant transcriptomic patterns in specific tissues, therefore the administration of disease-specific drugs, which could also induce anti-disease patterns, may exhibit enhanced efficacy when meeting the corresponding phase. To validate this point, in comprehensive consideration of the results of functional enrichment analysis, network centrality analysis and TAS comparative analysis, we prioritized the negative correlation between estrus and hypertension presented in aorta. In the next section, we experimentally explored whether the efficacy of anti-hypertensive drugs would be enhanced at the estrus phase *in vivo*.

### 3.4 Acebutolol and felodipine exhibit enhanced hypotensive effects in female rodents in estrus than in diestrus

**FIGURE 5:**
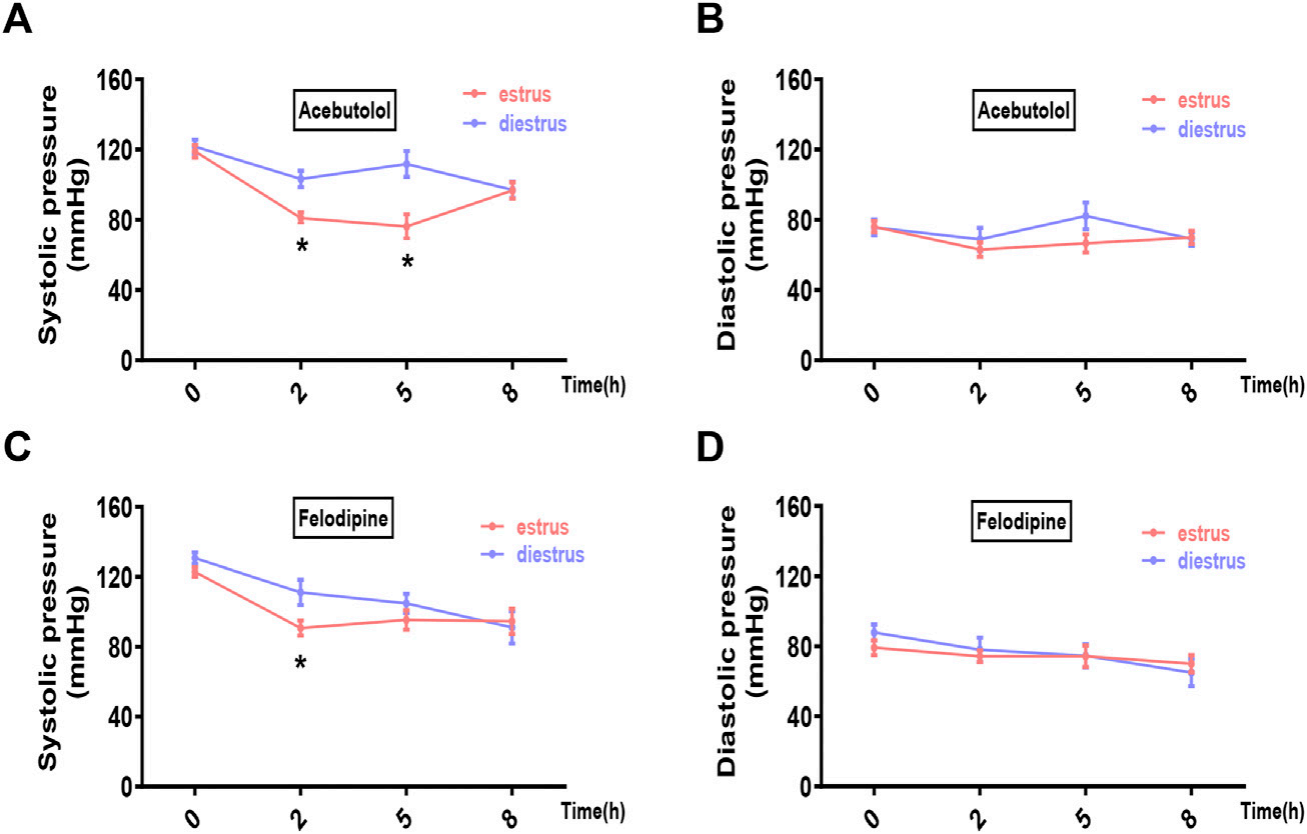
Acebutolol and felodipine exert stronger hypotensive effects on hypertensive mice in estrus than in diestrus. Hypertensive female mice were constructed *via* the subcutaneously infusion of angiotension-II for 2 weeks. Mice in estrus and diestrus were orally treated with acebutolol (2 mg/kg body weight) and felodipine (5 mg/kg body weight), respectively, and then blood pressure levels were monitored in the following 8 h (0, 2, 5, 8 h) **(A)** Systolic blood pressure and **(B)** diastolic blood pressure after acebutolol treatment **(C)** Systolic blood pressure and **(D)** diastolic blood pressure changes after felodipine treatment. *p*-values were determined by two-way ANOVA followed by Bonferroni correction, the values ≤0.05 were considered statistically significant. N = 8, *p-value ≤0.05.

**FIGURE 6:**
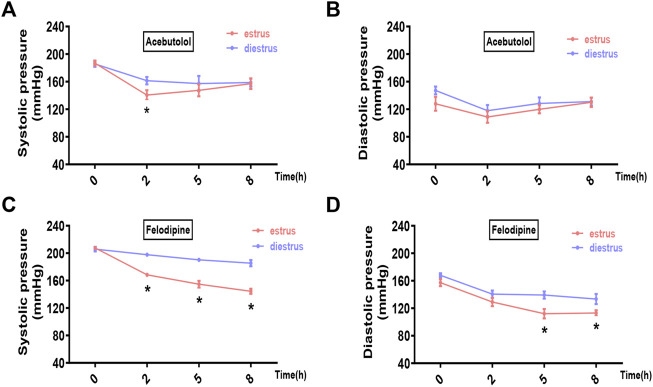
Acebutolol and felodipine exert stronger hypotensive effects on hypertensive rats in estrus than in diestrus. Female SHR rats in estrus and diestrus were orally treated with acebutolol (50 mg/kg body weight) and felodipine (30 mg/kg body weight), respectively, and then blood pressure levels were monitored in the following 8 h (0, 2, 5, 8 h) **(A)** Systolic blood pressure and **(B)** diastolic blood pressure changes after acebutolol treatment **(C)** Systolic blood pressure and **(D)** diastolic blood pressure changes after felodipine treatment. *p*-values were determined by two-way ANOVA followed by Bonferroni correction, the values ≤0.05 were considered statistically significant. N = 8, *p-value ≤0.05.

We chose two prevailing anti-hypertensive drugs, felodipine and acebutolol. Female hypertensive mice (constructed by angiotensin-II infusion) and rats (purchased SHR model) in estrus and diestrus were treated with one dose gavage of acebutolol and felodipine, respectively, and then the blood pressure levels were monitored in the following 8 h. As a result, both drugs are significantly active in lowering the systolic blood pressure of the hypertensive rodents in estrus than those in diestrus (Figures 5A, C for mice; Figures 6A, C for rats). As for the diastolic blood pressure, the drug administration also exhibits slightly stronger hypotensive effects in estrus (Figures 5B, D for mice; Figures 6B, D for rats), and the difference is prominently significant in the felodipine-SHR combination (Figure 6D). What’s more, the results are also repeatable in normotensive rodents (Supplementary Figures S11, S12). Therefore, it could be concluded that the hypotensive effects of acebutolol and felodipine are indeed enhanced in the female rodents in estrus than in diestrus, decently supporting our bioinformatics prediction.

## 4 Discussion

In this work, we generated a transcriptomic landscape covering 15 tissues dissected from estrus mice and diestrus mice and hereby conducted comprehensive analyses to investigate estrous cycle. Firstly, functional enrichment analysis of hDEGs shows that 10 tissues are responsive to the estrogen fluctuation between estrus and diestrus, reflecting the widespread influences of estrous cycle. Besides, neuro-functions, immuno-functions, blood coagulation and so on are also widely regulated, but the regulation could be mild except in vasculature because the hDEGs tend to be randomly distributed and even marginal in the molecular signaling network. To uncover the underlying mechanism of the transcriptional alteration, then we handpicked 13 key TFs of estrous cycle by means of the CIE tool, most of which can be supported by previous publications. Next, we carried out TAS comparative analysis to further characterize the changes of individual tissues during the estrous cycle. We firstly discussed about the reproductive tissues, namely ovary and uterus. The results are indicative of estrus’ and diestrus’ hallmarks, including estrogen stimulus, inflammation-like responses, ovulation and sexual impulse at the estrus phase as well as progesterone stimulus and fertile characteristics at the diestrus phase. Then we surveyed the other tissues and tried to recognize relatively beneficial and deleterious implications of estrus and diestrus. As a result, we discovered the PTSP phenomenon, where aorta, BAT and stomach are more protected by the estrus, while heart, liver, kidney, skeletal muscle, vein and WAT are more protected by the diestrus.

If the PTSP is homologous in human, then it is of great significance in woman precision medicine. Specifically, because different phases of reproductive cycle could result in different transcriptomic patterns (i.e., disease-resistant or disease-sensitive) in tissues, the possible phase-dependent drug efficacy should be considered in the clinical treatments towards pre-menopausal women. In this work, inspired by the prominent negative correlation between estrus and hypertension observed in aorta, we experimentally validated the synergy between estrus and two hypotensive drugs, felodipine and acebutolol, in both hypertensive and normotensive models. Therefore, to more precisely cure the pre-menopausal hypertensive patients, it may be proper to adaptively adjust the dosages of hypotensive drugs, where lower dosages during the peri-ovulatory period (which is homologous to estrus) are suggested to avoid possible hypotensive side-effects. It should be noted that although there exist differences in the post-ovulatory estrogen levels between human and rodents, their peri-ovulatory periods are both marked with nearly strongest estrogen stimulus at least, indicating similar physiological characteristics at the peri-ovulatory period. In addition to hypertension, the TAS comparative results also outline many other diseases such as arrhythmogenic cardiomyopathy, non-alcoholic fatty liver disease and type 2 diabetes. It could be speculated that the efficacy of the drugs for curing these diseases may be also fluctuant along during the reproductive cycle, which deserves to be further investigated in the future.

The PTSP phenomenon could also provide clues for the optimization of hormonal therapy (HT). It is well-known that the reproductive period/cycle could play a body protective role because the post-menopausal females bear significantly increased risks of CVDs, metabolic syndrome and so on than the pre-menopausal females in either human or animal models (Rosano et al., 2007; Maltais et al., 2009; Brady, 2015; Brooks et al., 2016; Muka et al., 2016; El Khoudary, 2017). The protection is considered to be closely associated with female hormones, especially estrogen, because peri- and post-menopause are featured with severe hormonal dysregulation and deficiency. Based on this principle, many HT clinical researches have been organized to investigate whether hormonal treatment is effective in post-menopausal disease prevention (Chester et al., 2018; El Khoudary et al., 2020). So far, some researches have revealed the efficiency of HT in preventing cardiovascular diseases in early menopausal women (Barrett-Connor and Bush, 1991; Hodis et al., 2016), but there are also researches reporting neutral effects (Hulley et al., 1998; Miller et al., 2019) and even risks of cardiovascular events and venous thrombosis (Straczek et al., 2005; Manson et al., 2013; Iorga et al., 2017). Retrospectively, previous HT studies focused more on estrogen while progesterone only serves as an estrogen antagonist (Chester et al., 2018; El Khoudary et al., 2020). However, our results also underline the importance of progesterone because quite a few tissues, especially heart and vein, which have been shown to bear risks in estrogen-centric HT researches (Straczek et al., 2005; Manson et al., 2013; Iorga et al., 2017), are more protected by the progesterone-abundant diestrus. Furthermore, in consideration of the asynchronous protective effects on different tissues during the reproductive cycle, the overall protection provided by reproductive period is plausibly achieved by the dynamic equilibrium resulting from the alternated hormonal milieus. However, in previous HT studies, the components and daily dosages of hormonal treatment are always constant (Chester et al., 2018; El Khoudary et al., 2020). Therefore, to further optimize HT, it may be helpful to pay more attention on the progesterone and adjust the conventional scheme of constant hormonal treatment.

Nevertheless, there are still limitations in this work. For example, to approximately capture the transcriptomic changes, we only assigned triplicate for each tissue-phase group, which may be less powerful to control outliers. Although the samples are well-clustered (Figure 1B), it is still necessary to set more replicates in future in-depth researches. Besides, in the TAS comparative analysis, the genes with small fold change in the curated TASs may be randomly distributed, thus would disturb the SCCs and result in some false negative results. With the improvement of gene functional annotations, this issue could be properly addressed in the future by building TASs only considering the genes with clear physiological or pathological relations instead of the whole transcriptome. Finally, an important hypothesis of this work is that the human menstrual cycle is homologous to the rodent estrous cycle. However, there are still physiological differences between them. For example, the rodent estrous cycle only spends 4–5 days, but the human menstrual cycle lasts for about 1 month. Besides, in human, the corpus luteum is capable of secreting estrogen, therefore the estrogen level will slightly rise up again after ovulation, but the phenomenon is absent in rodents. These differences may hinder the generalization from the basic researches on female rodents to the clinical applications on women. Therefore, this work could only provide plausible clues for developing precision medicine strategies for pre-menopausal women, and extensive clinical researches are still required in the future.

## Publisher’s note

All claims expressed in this article are solely those of the authors and do not necessarily represent those of their affiliated organizations, or those of the publisher, the editors and the reviewers. Any product that may be evaluated in this article, or claim that may be made by its manufacturer, is not guaranteed or endorsed by the publisher.

## Data Availability

The datasets presented in this study can be found in online repositories. The names of the repository/repositories and accession number(s) can be found below: Gene Expression Omnibus, accession number: GSE131172.

## Supplementary material

Supplementary Table S1 Supplementary Data S1. Functional transcription factors identified by CIE.

Supplementary Table S2 Supplementary Data S2. Details of manually curated transcriptomic alteration signatures.

Supplementary Data Sheet S1 Supplementary Text and Figures.
