# Supplementary material for: A multi-tissue transcriptomic landscape of female mice in estrus and diestrus provides clues for precision medicine
Source: Front Cell Dev Biol. 2022 Dec 16;10:983712. doi: 10.3389/fcell.2022.983712 (PMC9800588; doi:10.3389/fcell.2022.983712)
Supplement: Supplementary file 2 [file DataSheet1.pdf]

Supplementary Material

1 Supplementary Text

1.1 The effects of estrous cycle on liver and adipose inferred from TAS comparative results

In liver, the estrus-induced TAS are clearly positively correlated with the TASs of non-alcoholic fatty liver (SCC = 0.250, p-value = 2.05e-168), liver infection (West Nile virus infection: SCC = 0.164, p-value = 2.40e-54; Visceral leishmaniasis infection: SCC = 0.161, p-value = 2.29-80; etc.), liver cancer (MycTg + KrasG12D model: SCC = 0.185, p-value = 1.01e-80; Pten-/- Shp2-/- model: SCC = 0.163, p-value = 1.53e-55; etc.) and so on, while tend to be negatively correlated with the beneficial TASs of caloric restriction (SCC = -0.156, p-value = 4.07e-81) and disease therapies (Supplementary Figure S7A). Previous studies have revealed that estrogen protects liver from hepatic steatosis, hepatitis, hypercholesterolemia and so on (Evans et al., 2002; Takeda et al., 2003; Lemieux et al., 2005), but in our results, diestrus, which is featured with progesterone, is more protective to liver relative to estrus, which is featured with estrogen. Unfortunately, few studies have reported progesterone's protectiveness to liver and more inconsistently, progesterone treatment is even shown to exacerbate drug-induced liver injury (Toyoda et al., 2011). One possible explanation is that, unlike the progesterone-treated liver, the liver in diestrus has underwent the estrogen stimulus before, so there may exist an unknown cross-time interaction between estrogen and progesterone which could produce more beneficial effects on liver. This hypothesis is required to be further investigated.

In BAT, estrus mimics many beneficial processes such as WAT browning (CL316243 treatment induced: SCC = 0.211, p-value = 2.64e-140; Egr1-/- model: SCC = 0.127, p-value = 3.91e-55), caloric restriction (SCC = 0.158, p-value = 6.35e-83), physical exercise (SCC = 0.118, p-value = 1.98e-38) and so on, meanwhile tends to negatively correlate with deleterious TASs of insulin resistance (Crls1-/- model: SCC = -0.251, p-value = 2.98e-200; IR+/- IRS1+/- model: SCC = -0.174, p-value = 2.39e-97), obesity (SCC = -0.225, p-value = 1.83e-167), type 2 diabetes (SCC = -0.102, p-value = 8.98e-34) and the like, exhibiting a salutary BAT activating pattern (Supplementary Figure S7B). The activation of BAT has anti-obesity effects because it is capable of enhancing metabolic rate and accelerating WAT consumption (Boon and van Marken Lichtenbelt, 2016). However, a clear obesity-sensitive pattern unexpectedly presents in the estrus-induced TAS of WAT (Supplementary Figure S7C). Therefore, estrous cycle's effects on adipose tissues could be sophisticated where BAT gets more benefits from the estrus, whereas WAT gets more benefits from the diestrus. The opposition deserves to be deeply investigated.

1.2 The effects of estrous cycle on other tissues inferred from TAS comparative results

In kidney, skeletal muscle and stomach, the TAS comparative results are less obvious to exhibit beneficial and deleterious implications, but from the sunburst plots, visible tendencies can be observed when comparing with background, where stomach is more protected by the estrus, while kidney and skeletal muscle are more protected by the diestrus (Supplementary Figure S8).

As for the others tissues, namely cerebrum, duodenum, hypothalamus and spleen, the TAS comparative results are ambiguous and even the sunburst plots are less informative to reflect beneficial and deleterious effects (Supplementary Figure S9-S10). This may be attributed to two

reasons: (1) The transcriptomic changes of these tissues during the estrous cycle may be indeed irregular. (2) Because these tissues are with extraordinarily complex biomechanisms, it may be less powerful to identify beneficial and deleterious implications only from the bulk transcriptomic alteration data. If so, the single cell sequencing technique may be efficient to profile more detailed changes of these tissues and is supposed to be considered in the future.

References

Boon, M.R., and van Marken Lichtenbelt, W.D. (2016). Brown Adipose Tissue: A Human Perspective. *Handb Exp Pharmacol* 233**,** 301-319. doi: 10.1007/164_2015_11.

Evans, M.J., Lai, K., Shaw, L.J., Harnish, D.C., and Chadwick, C.C. (2002). Estrogen receptor alpha inhibits IL-1beta induction of gene expression in the mouse liver. *Endocrinology* 143(7)**,** 2559-2570. doi: 10.1210/endo.143.7.8919.

Lemieux, C., Phaneuf, D., Labrie, F., Giguere, V., Richard, D., and Deshaies, Y. (2005). Estrogen receptor alpha-mediated adiposity-lowering and hypocholesterolemic actions of the selective estrogen receptor modulator acolbifene. *Int J Obes (Lond)* 29(10)**,** 1236-1244. doi: 10.1038/sj.ijo.0803014.

Takeda, K., Toda, K., Saibara, T., Nakagawa, M., Saika, K., Onishi, T., et al. (2003). Progressive development of insulin resistance phenotype in male mice with complete aromatase (CYP19) deficiency. *J Endocrinol* 176(2)**,** 237-246. doi: 10.1677/joe.0.1760237.

Toyoda, Y., Miyashita, T., Endo, S., Tsuneyama, K., Fukami, T., Nakajima, M., et al. (2011). Estradiol and progesterone modulate halothane-induced liver injury in mice. *Toxicol Lett* 204(1)**,** 17-24. doi: 10.1016/j.toxlet.2011.03.031.

2 Supplementary Figures

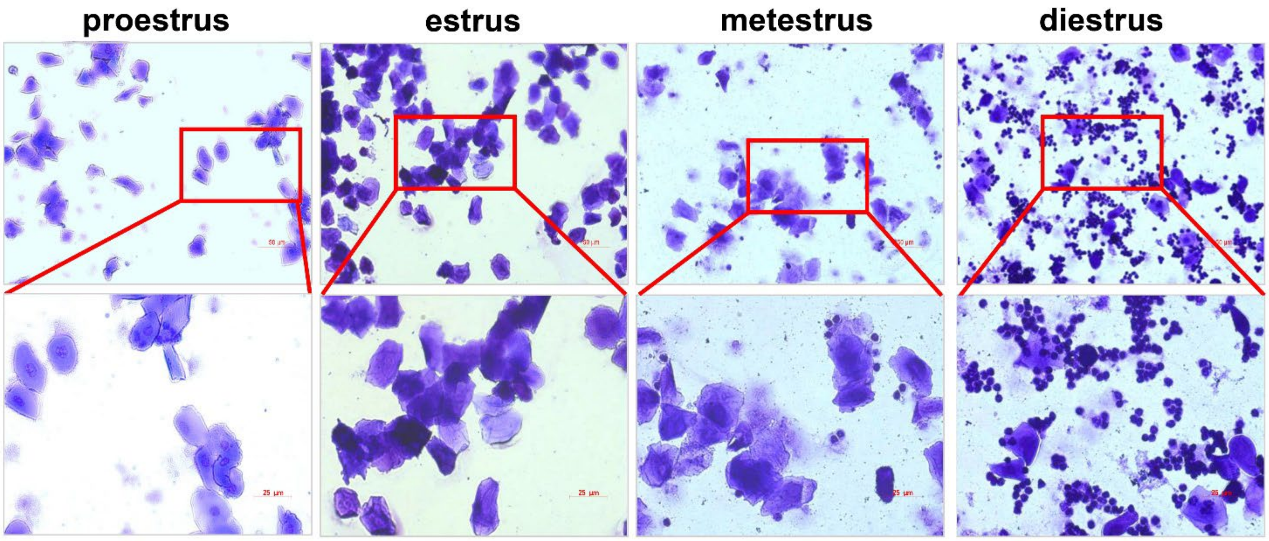

Supplementary Figure S1. Characterization of estrous cycle in female C57BL/6J mice. Representative images of methylene blue staining for mice vaginal epithelial cells in different stages: proestrus, nucleated epithelial cells; estrus, nucleated cornified cells; metestrus, consisting of leukocytes, cornified, and nucleated epithelial cells; and diestrus, predominantly of leucocytes, partly nucleated epithelial cells. N = 9. Scale bar: 50µm or 25µm.

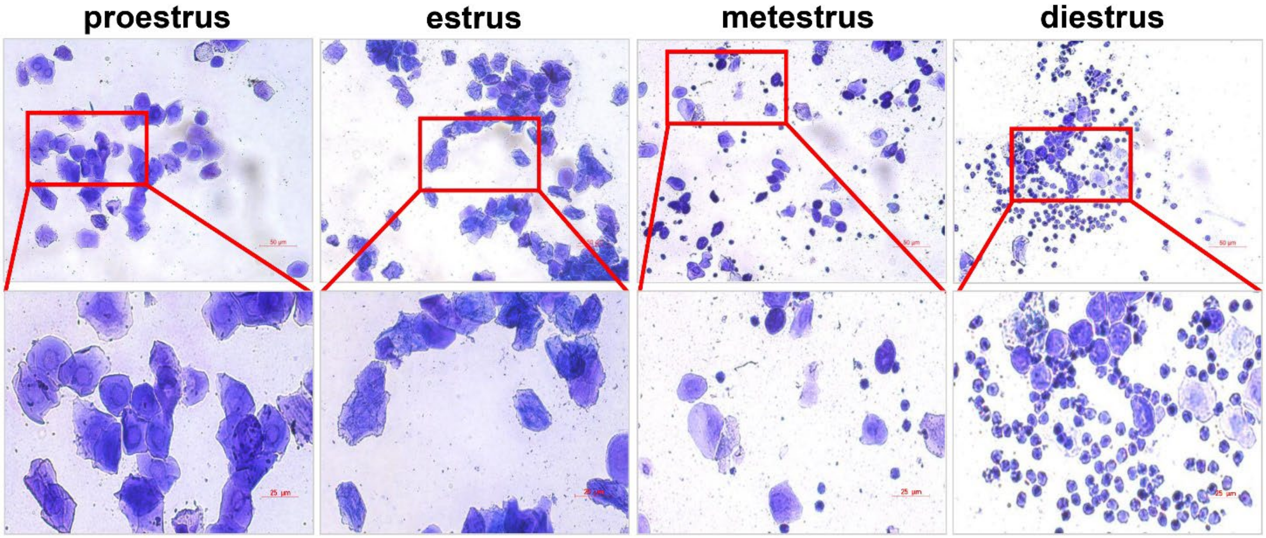

Supplementary Figure S2. Characterization of estrous cycle in female SD rats. Representative photomicrographs of vaginal images from female rats in four stages. N = 6. Scale bar: 50μm or 25μm.

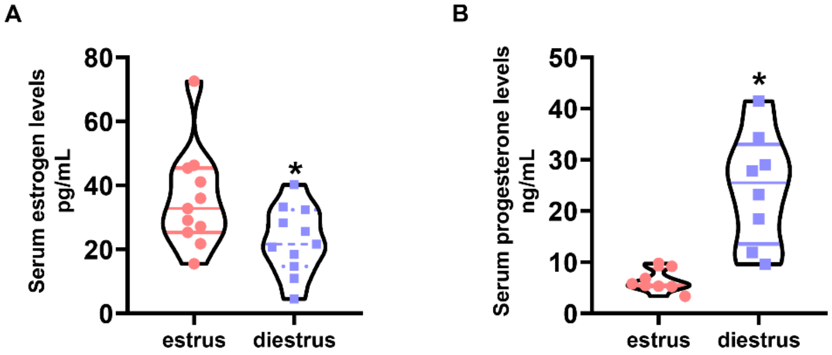

Supplementary Figure S3. Serum estrogen and progesterone levels of mice in estrus and diestrus. The internal canthus blood of mice was collected using heparinized capillaries tube. All samples were placed at room temperature for 2 hours followed by centrifugation for 20 minutes at 1000 × g (4℃). Serum estrogen and progesterone levels were quantified using Elisa Kits. (A) Serum estrogen levels of mice in estrus and diestrus. N = 11. (B) Serum progesterone levels of mice in estrus and diestrus. P-values were calculated by unpaired t-test. N = 8. *p-value ≤ 0.05.

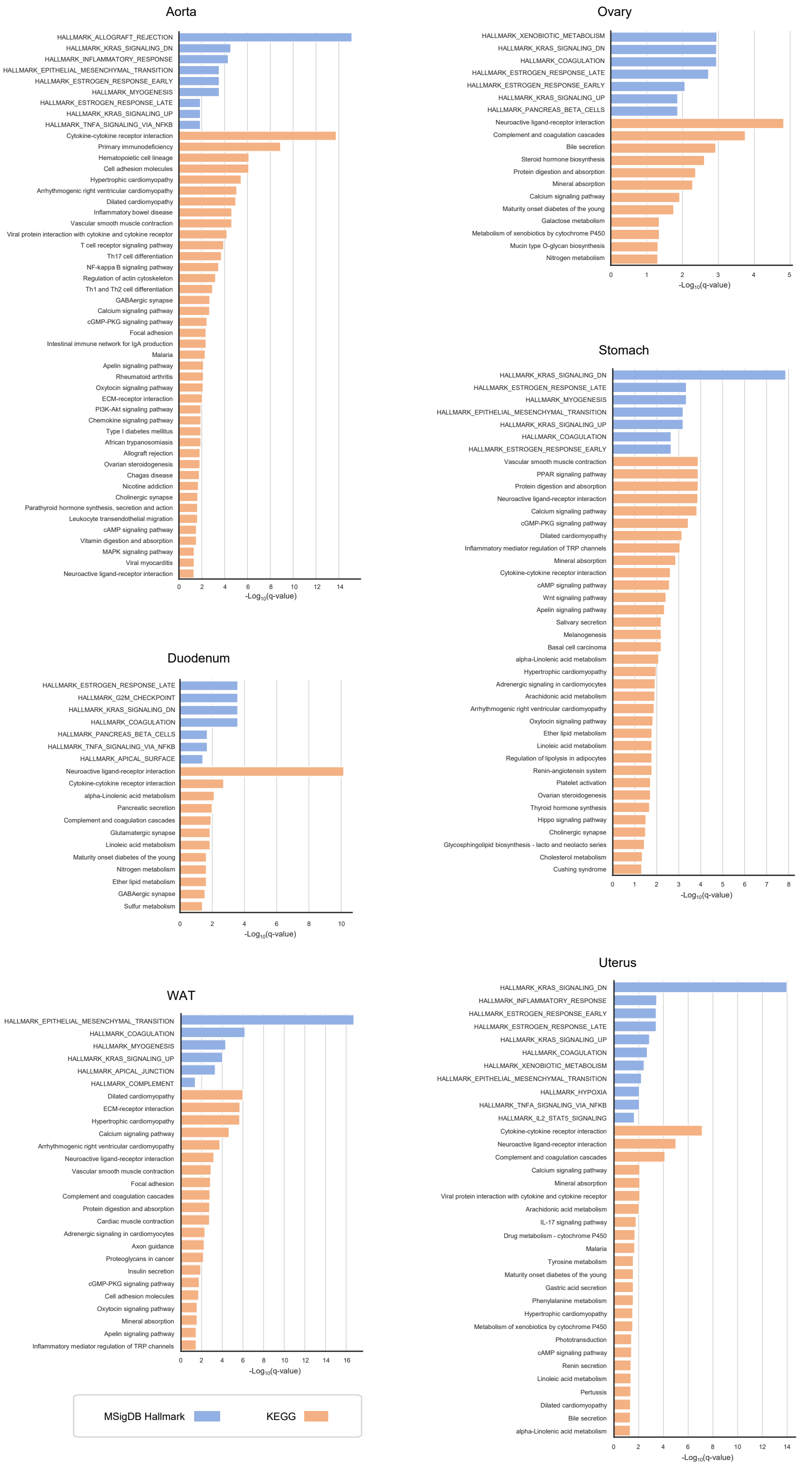

Supplementary Figure S4. KEGG and MSigDB Hallmark functional enrichment results of ECTTs.

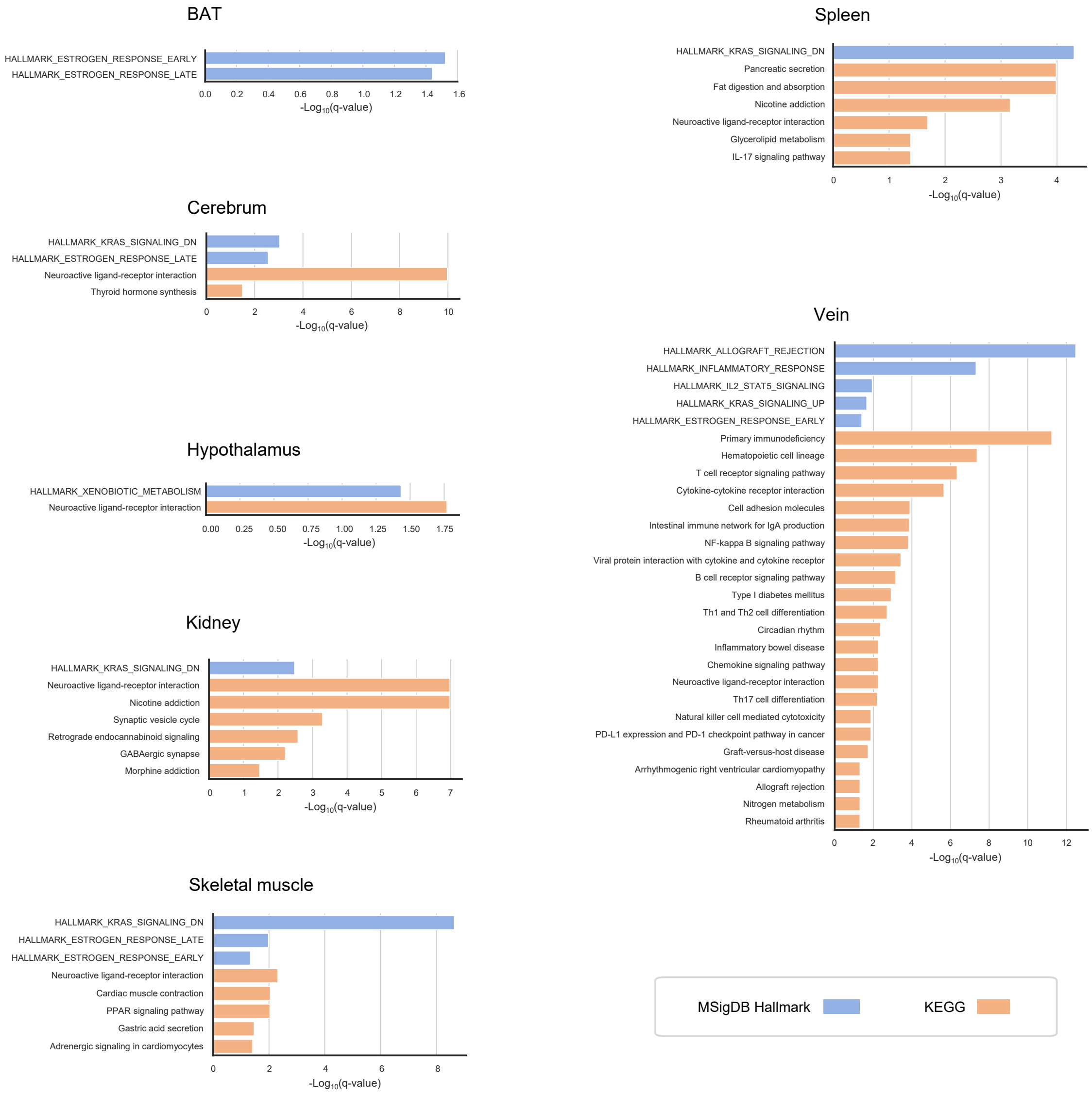

Supplementary Figure S5. KEGG and MSigDB Hallmark functional enrichment results of non-ECTTs.

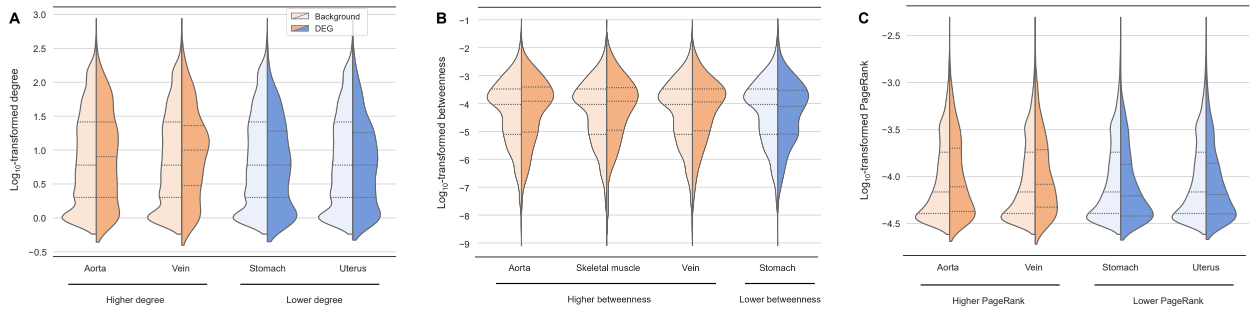

Supplementary Figure S6. The tissues whose hDEGs are of significantly higher or lower hubness in comparison with background in the molecular signaling network. Gene hubness was measured by three network centrality indices: (A) degree, (B) betweenness and (C) PageRank. The left half and the right half violin plot respectively show the hubness distribution of background and hDEGs, and the dash lines represent quartiles. The centrality indices are log_{10}-transformed.

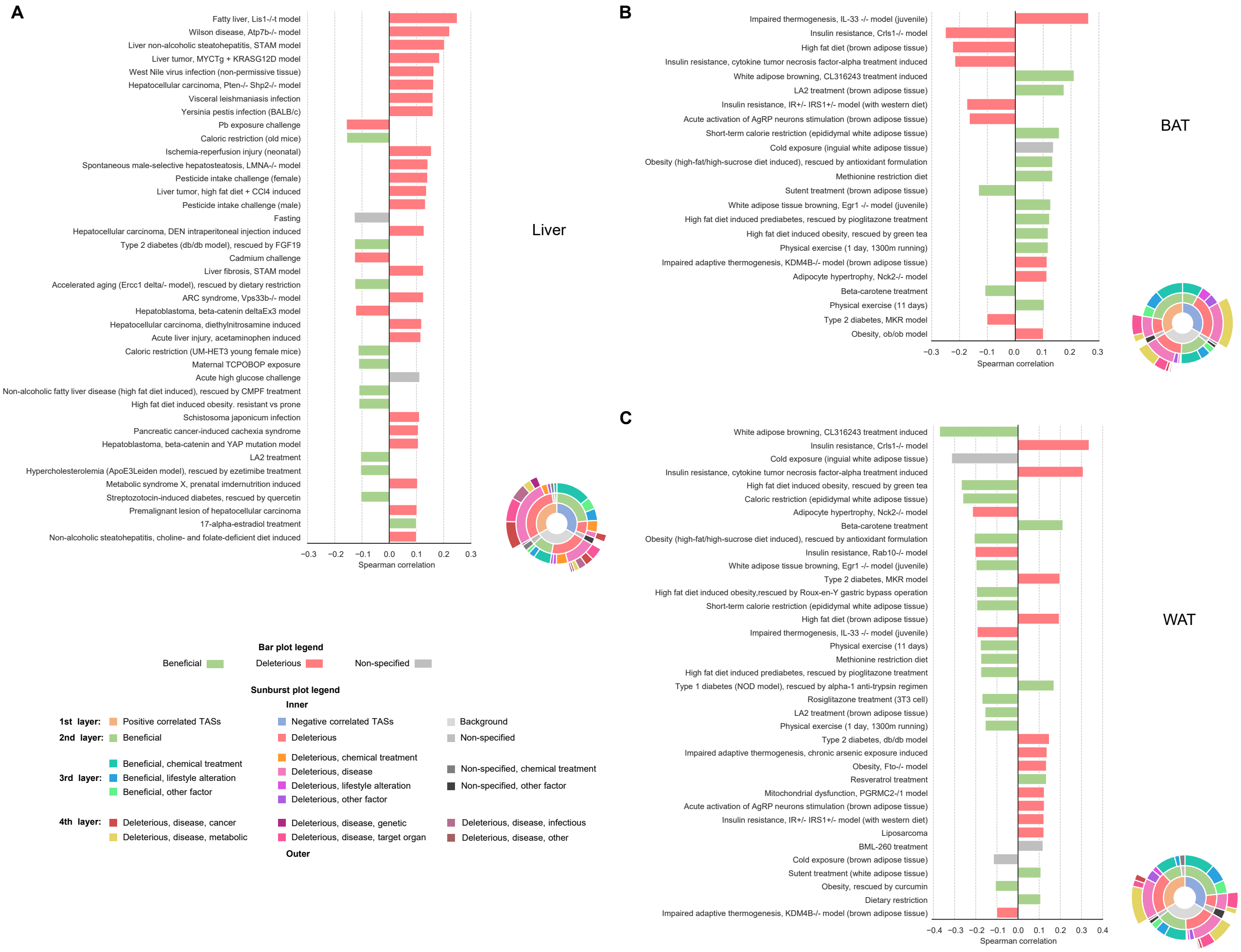

Supplementary Figure S7. Significantly correlated relationships between estrus-induced TASs and curated TASs in liver and adipose tissues. Significantly correlated relationships in (A) liver, (B) brown adipose tissue and (C) white adipose tissue.

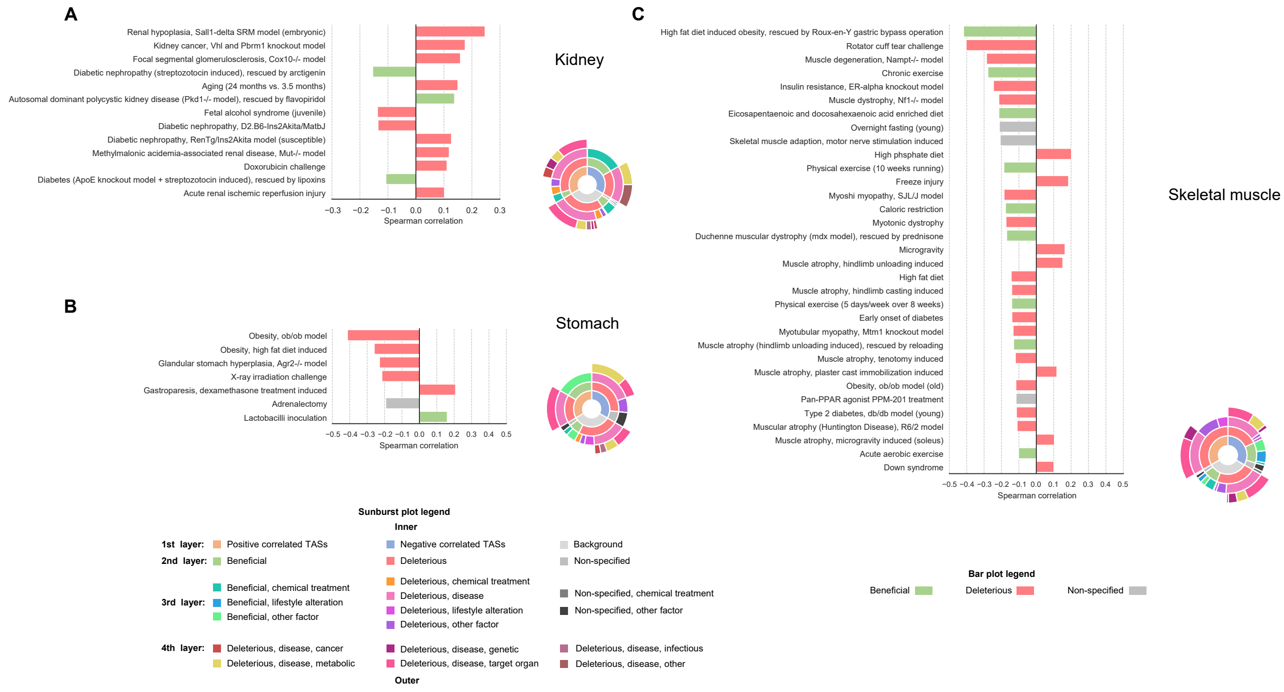

Supplementary Figure S8. Significantly correlated relationships between estrus-induced TASs and curated TASs in kidney, stomach and skeletal muscle. Significantly correlated relationships in (A) kidney, (B) stomach and (C) skeletal muscle.

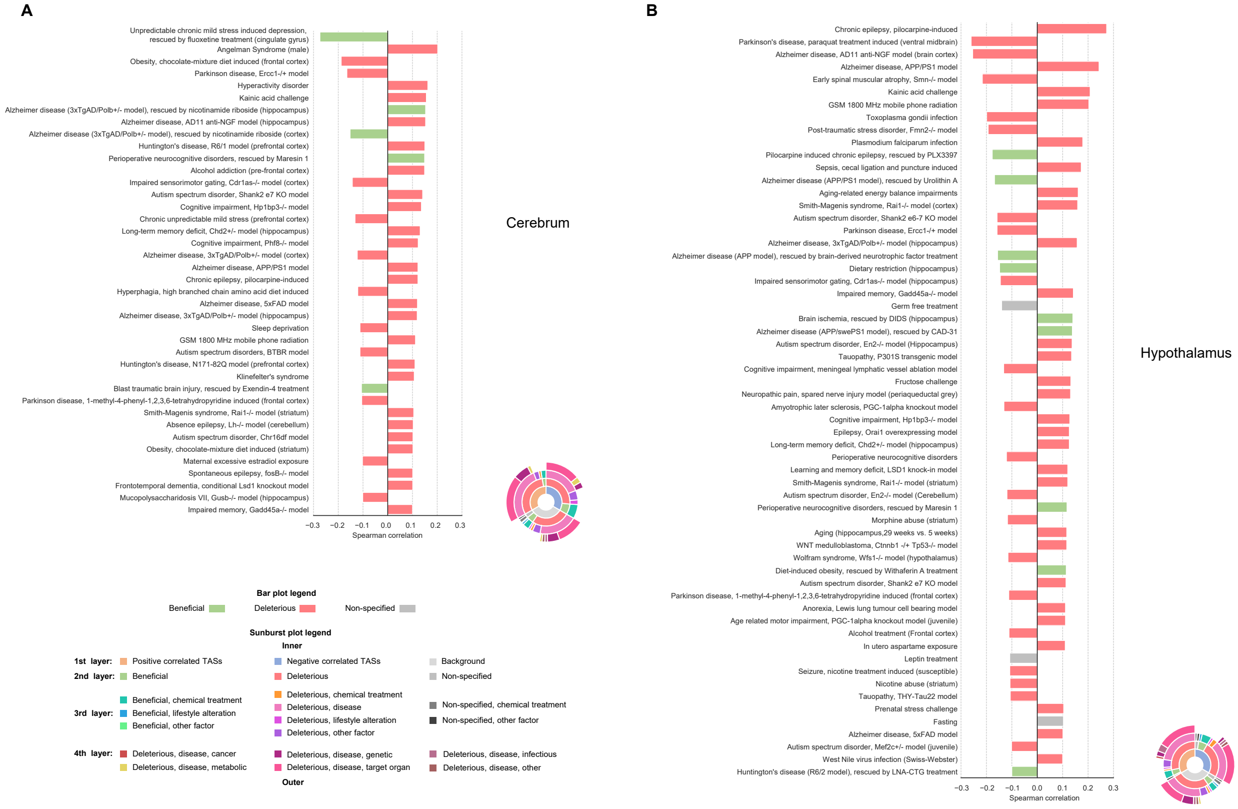

Supplementary Figure S9. Significantly correlated relationships between estrus-induced TASs and curated TASs in cerebrum and hypothalamus. Significantly correlated relationships in (A) cerebrum and (B) hypothalamus.

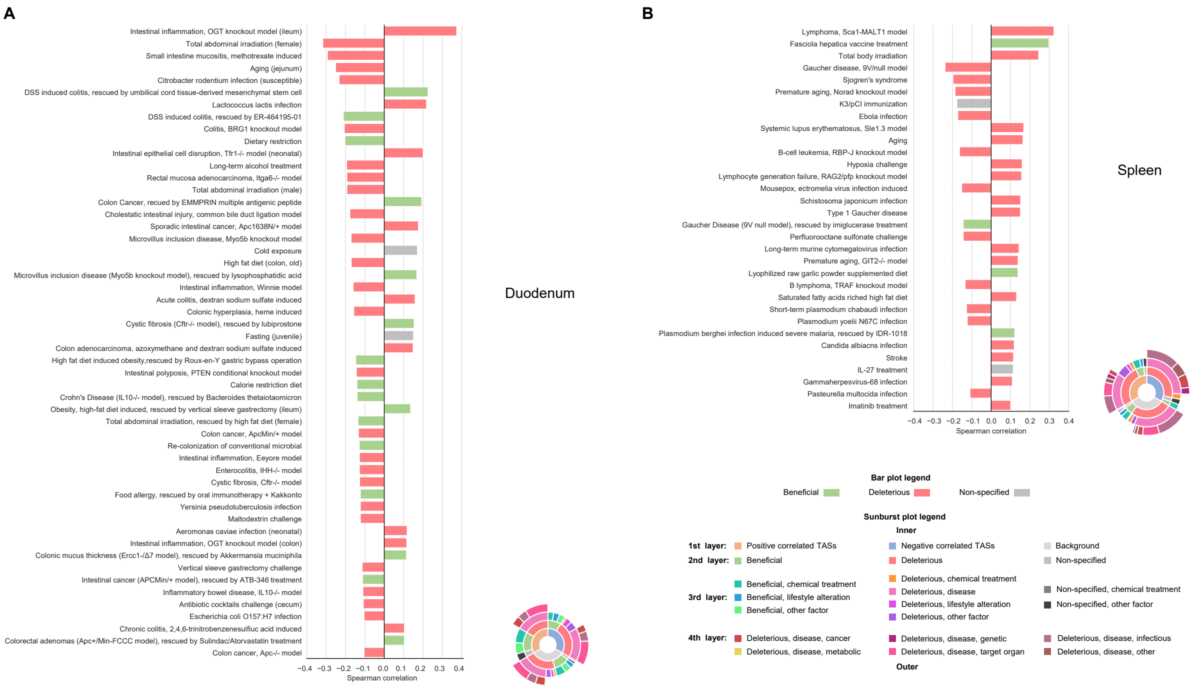

Supplementary Figure S10. Significantly correlated relationships between estrus-induced TASs and curated TASs in duodenum and spleen. Significantly correlated relationships in (A) duodenum and (B) spleen.

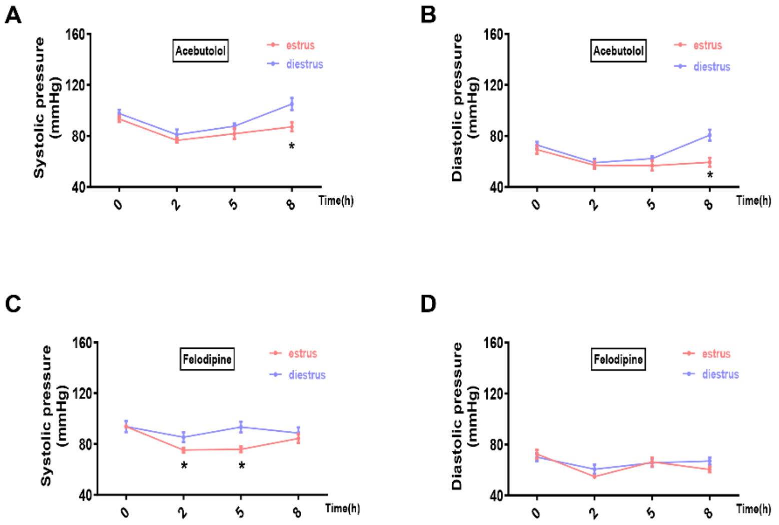

Supplementary Figure S11. Effects of hypotensive drugs (acebutolol and felodipine) on normotensive mice in estrus and diestrus. Normotensive female C57BL/6 mice in estrus and diestrus were gavaged with 2mg/kg body weight acebutolol and 5mg/kg body weight felodipine. The blood pressure levels were monitored in the following 8 hours (0h, 2h, 5h, 8h) after administration using tail-cuff method. (A) Systolic blood pressure and (B) diastolic blood pressure changes after acebutolol treatment. (C) Systolic blood pressure and (D) diastolic blood pressure changes after felodipine treatment. P-values were determined by two-way ANOVA followed by Bonferroni correction, values ≤ 0.05 were considered statistically significant. N = 9, *p-value ≤ 0.05.

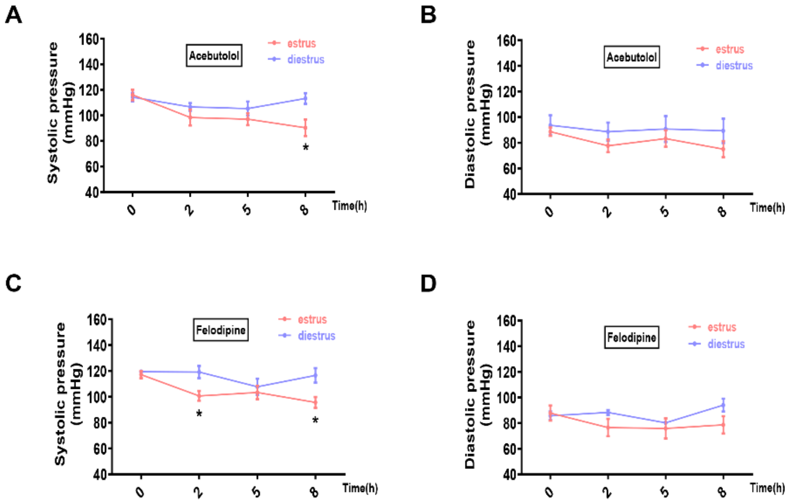

Supplementary Figure S12. Effects of hypotensive drugs (acebutolol and felodipine) on normotensive rats in estrus and diestrus. Normotensive female SD rats in estrus and diestrus were gavaged with 50mg/kg body weight acebutolol and 30mg/kg body weight felodipine, respectively. The blood pressure levels were monitored in the following 8 hours (0h, 2h, 5h, 8h) after administration using tail-cuff method. (A) Systolic blood pressure and (B) diastolic blood pressure changes after acebutolol treatment. (C) Systolic blood pressure and (D) diastolic blood pressure changes after felodipine treatment. P-values were determined by two-way ANOVA followed by Bonferroni correction, values ≤ 0.05 were considered statistically significant. N = 6, * p-value ≤ 0.05.
